# Supplementary material for: Utilization of adequately iodized salt and its associated factors in Tanzania rural areas: a case of Kilwa district, Lindi region, 2023
Source: PLoS One. 2025 Nov 24;20(11):e0337337. doi: 10.1371/journal.pone.0337337 (PMC12643270; doi:10.1371/journal.pone.0337337)
Supplement: Attachment — Submitted filename: PONE-D-25-02467-review.docx [file pone.0337337.s002.docx]

**PLOS ONE**

### Utilization of adequately iodized salt and its associated factors in Tanzania rural areas: a case of Kilwa district, Lindi region, 2023

--Manuscript Draft--

| **Manuscript Number:** | PONE-D-25-02467 |
| --- | --- |
| **Article Type:** | Research Article |
| **Full Title:** | Utilization of adequately iodized salt and its associated factors in Tanzania rural areas: a case of Kilwa district, Lindi region, 2023 |
| **Short Title:** | Utilization of iodized salt and associated factors in Kilwa district, Tanzania, 2023 |
| **Corresponding Author:** | George Mrema, MD, MSc  Ministry of Health and Community Service: Ministry of Health Community Development Gender Elderly and Children  DODOMA, TANZANIA, UNITED REPUBLIC OF |
| **Keywords:** | Iodized salt, Iodine deficiency, Utilization, Rural Tanzania |
| **Abstract:** | Background: Utilization of salt with adequate iodine content is crucial for prevention of iodine deficiency disorders. However, utilization of adequately iodized salt in Tanzania regions varies and the situation is worse in areas with local salt production sites.  Hence, this study aimed to assess household-level utilization of adequately iodized salt and its associated factors in Kilwa district, Tanzania.  Methodology: A cross-sectional research design was used in this study. A total of 493 households were systematically sampled to join the study and one-on-one interview was conducted to the head of each household. Purposive sampling was used to recruit 14 local salt producers where in-depth interviews were conducted to them. Iodine rapid test was performed onsite followed by iodine content analysis at Kigoma Regional Referral Hospital (RRH) laboratory. Quantitative data were analyzed using STATA while NVivo software was applied for analysis of qualitative data. Association between dependent and independent variables was assessed by binary logistic regression.  Result: Only 9.4% of households utilized adequately iodized salt. Family size with equal or less than five members (Adjusted Odds Ratio (AOR), 3.49; 95% CI: 1.62, 7.54), good knowledge about iodized salt (AOR. 4.97; 95% CI: 2.04, 12.11), salt storage in dry area (AOR, 4.44; 95% CI: 1.51, 13.07), expose of salt to sunlight (AOR, 0.29; 95% CI: 0.10, 0.85) and staying with salt for less than two months (AOR, 2.34; 95% CI: 1.10, 5.00) were significantly associated with utilization of adequately iodized salt. Poor protection of salt at the production sites, supply of non-iodized salt to workers, unavailability of training to local salt producers, presence of other local salt producers in the community and community preference on non-iodized salt were the reasons for low availability of iodized salt in Kilwa district as reported by local salt producers.  Conclusion: The prevalence of utilization of adequately iodized salt in Kilwa district is very low. Family size, knowledge about iodized salt, storage place, exposure to sunlight and salt stay time were associated with utilization of adequately iodized salt. Local salt producers contribute a lot to poor availability of iodized salt. It is recommended among others that Tanzania Government should put more efforts on awareness campaigns to raise knowledge in the study area on benefits of using iodized salt. |
| **Order of Authors:** | David Mahwera |
|  | Rose Msaki |
|  | Rogath Kishimba |
|  | Fatma Abdallah |
|  | Danford Mahwera |
|  | Vicent Assay |
|  | George Mrema, MD, MSc |
|  | Geofrey Mchau |

*Powered by Editorial Manager® and ProduXion Manager® from Aries Systems Corporation*

|  | Germana Leyna |
| --- | --- |
|  | Theresia Ambrose |
| **Additional Information:** |  |
| **Question** | **Response** |
| **Financial Disclosure**  Enter a financial disclosure statement that describes the sources of funding for the work included in this submission. Review the [submission guidelines](http://journals.plos.org/plosone/s/submission-guidelines#loc-financial-disclosure-statement) for detailed requirements. View published research articles from [*PLOS ONE*](https://journals.plos.org/plosone/) for specific examples.  This statement is required for submission and **will appear in the published article** if the submission is accepted. Please make sure it is accurate. | Yes |
| **Funded studies**  Enter a statement with the following details:   - Initials of the authors who received each award - Grant numbers awarded to each author - The full name of each funder - URL of each funder website - Did the sponsors or funders play any role in the study design, data collection and analysis, decision to publish, or preparation of the manuscript? |  |
| Did you receive funding for this work? |  |
| Please add funding details.  as follow-up to "**Financial Disclosure**  Enter a financial disclosure statement that describes the sources of funding for the work included in this submission. Review the [submission guidelines](http://journals.plos.org/plosone/s/submission-guidelines#loc-financial-disclosure-statement) for detailed requirements. View published research articles from [*PLOS ONE*](https://journals.plos.org/plosone/) for specific examples.  This statement is required for submission and **will appear in the published article** if the submission is accepted. Please make | This work is supported by the United States President’s Emergency Plan for AIDS Relief (PEPFAR) through the Centre of Excellence in Health Monitoring and Evaluation, Mzumbe University under the U.S Centers for Disease Control and Prevention (CDC), Project Cooperative Agreement No: NU2GGH002292. Additionally, they provide support in the training of residents in Tanzania's Field Epidemiology and Laboratory Training Program. |

*Powered by Editorial Manager® and ProduXion Manager® from Aries Systems Corporation*

| sure it is accurate. |  |
| --- | --- |
| **Funded studies**  Enter a statement with the following details:   - Initials of the authors who received each award - Grant numbers awarded to each author - The full name of each funder - URL of each funder website - Did the sponsors or funders play any role in the study design, data collection and analysis, decision to publish, or preparation of the manuscript? |  |
| Did you receive funding for this work?" |  |
| Please select the country of your main research funder (please select carefully as in some cases this is used in fee calculation).  as follow-up to "**Financial Disclosure**  Enter a financial disclosure statement that describes the sources of funding for the work included in this submission. Review the [submission guidelines](http://journals.plos.org/plosone/s/submission-guidelines#loc-financial-disclosure-statement) for detailed requirements. View published research articles from [*PLOS ONE*](https://journals.plos.org/plosone/) for specific examples.  This statement is required for submission and **will appear in the published article** if the submission is accepted. Please make sure it is accurate. | UNITED STATES - US |
| **Funded studies**  Enter a statement with the following details:   - Initials of the authors who received each award - Grant numbers awarded to each author - The full name of each funder - URL of each funder website - Did the sponsors or funders play any role in the study design, data collection and analysis, decision to publish, or preparation of the manuscript? |  |
| Did you receive funding for this work?" |  |

*Powered by Editorial Manager® and ProduXion Manager® from Aries Systems Corporation*

| **Competing Interests**  Use the instructions below to enter a competing interest statement for this submission. On behalf of all authors, disclose any [competing interests](http://journals.plos.org/plosone/s/competing-interests) that could be perceived to bias this work—acknowledging all financial support and any other relevant financial or non- financial competing interests.  This statement is required for submission and **will appear in the published article** if the submission is accepted. Please make sure it is accurate and that any funding sources listed in your Funding Information later in the submission form are also declared in your Financial Disclosure statement.  View published research articles from  [*PLOS ONE*](http://journals.plos.org/plosone/) for specific examples. | The authors have declared that no competing interests exist |
| --- | --- |
| **NO authors have competing interests**  Enter: *The authors have declared that no competing interests exist.*  **Authors with competing interests**  Enter competing interest details beginning with this statement:  *I have read the journal's policy and the authors of this manuscript have the following competing interests: [insert competing interests here]* |  |
| * typeset |  |
| **Ethics Statement**  Enter an ethics statement for this submission. This statement is required if the study involved:   - Human participants - Human specimens or tissue - Vertebrate animals or cephalopods | Ethical approval was obtained from the ethical review board at Muhimbili University of Health and Allied Sciences with approval number MUHAS-REC-11-2022-1462.  Participants were given explanation on the objectives of the study then written and verbal informed consent was given to the participants; only consented participants were involved in the study. Name and other personal identifiers were excluded in the questionnaire and only identification numbers were used instead to ensure confidentiality. Permission to enter into selected wards was obtained from Ward Executive Officers (WEOs). |

*Powered by Editorial Manager® and ProduXion Manager® from Aries Systems Corporation*

| - Vertebrate embryos or tissues - Field research   Write "N/A" if the submission does not require an ethics statement.  General guidance is provided below. Consult the [submission guidelines](http://journals.plos.org/plosone/s/submission-guidelines#loc-guidelines-for-specific-study-types) for detailed instructions. **Make sure that all information entered here is included in the Methods section of the manuscript.** |  |
| --- | --- |

*Powered by Editorial Manager® and ProduXion Manager® from Aries Systems Corporation*

| **Format for specific study types**  **Human Subject Research (involving human participants and/or tissue)**   - Give the name of the institutional review board or ethics committee that approved the study - Include the approval number and/or a statement indicating approval of this research - Indicate the form of consent obtained (written/oral) or the reason that consent was not obtained (e.g. the data were analyzed anonymously)   **Animal Research (involving vertebrate animals, embryos or tissues)**   - Provide the name of the Institutional Animal Care and Use Committee (IACUC) or other relevant ethics board that reviewed the study protocol, and indicate whether they approved this research or granted a formal waiver of ethical approval - Include an approval number if one was obtained - If the study involved *non-human primates*, add *additional details* about animal welfare and steps taken to ameliorate suffering - If anesthesia, euthanasia, or any kind of animal sacrifice is part of the study, include briefly which substances and/or methods were applied   **Field Research**  Include the following details if this study involves the collection of plant, animal, or other materials from a natural setting:   - Field permit number - Name of the institution or relevant body that granted permission |  |
| --- | --- |
|  |  |
| **Data Availability**  Authors are required to make all data underlying the findings described fully available, without restriction, and from the time of publication. PLOS allows rare exceptions to address legal and ethical concerns. See the [PLOS Data Policy](http://journals.plos.org/plosone/s/data-availability) and [FAQ](http://journals.plos.org/plosone/s/data-availability#loc-faqs-for-data-policy) for detailed information. | Yes - all data are fully available without restriction |

*Powered by Editorial Manager® and ProduXion Manager® from Aries Systems Corporation*

| A Data Availability Statement describing where the data can be found is required at submission. Your answers to this question constitute the Data Availability Statement and **will be published in the article**, if accepted. |  |
| --- | --- |
| **Important:** Stating ‘data available on request from the author’ is not sufficient. If your data are only available upon request, select ‘No’ for the first question and explain your exceptional situation in the text box. |  |
| Do the authors confirm that all data underlying the findings described in their manuscript are fully available without restriction? |  |
| **Describe where the data may be found in full sentences. If you are copying our sample text, replace any instances of XXX with the appropriate details.**   - If the data are **held or will be held in a public repository**, include URLs, accession numbers or DOIs. If this information will only be available after acceptance, indicate this by ticking the box below. For example: *All XXX files are available from the XXX database (accession number(s) XXX, XXX.*). - If the data are all contained **within the manuscript and/or Supporting Information files**, enter the following: *All relevant data are within the manuscript and its Supporting Information files.* - If neither of these applies but you are able to provide **details of access elsewhere**, with or without limitations, please do so. For example:   *Data cannot be shared publicly because of [XXX]. Data are available from the XXX Institutional Data Access / Ethics Committee (contact via XXX) for researchers who meet the criteria for access to confidential data.*  *The data underlying the results presented in the study are available from (include the name of the third party* | All relevant data are within the manuscript and its Supporting Information files. |

*Powered by Editorial Manager® and ProduXion Manager® from Aries Systems Corporation*

| *and contact information or URL).*   - This text is appropriate if the data are owned by a third party and authors do not have permission to share the data.   * typeset |  |
| --- | --- |
| Additional data availability information: |  |

*Powered by Editorial Manager® and ProduXion Manager® from Aries Systems Corporation*

Manuscript	[Click here to access/download;Manuscript;Manuscript.docx](https://www2.cloud.editorialmanager.com/pone/download.aspx?id=38961702&guid=38c5715a-d8c1-4797-b234-2118360117d3&scheme=1)[<https://www2.cloud.editorialmanager.com/pone/download.aspx?id=38961702&guid=38c5715a-d8c1-4797-b234-2118360117d3&scheme=1>](https://www2.cloud.editorialmanager.com/pone/download.aspx?id=38961702&guid=38c5715a-d8c1-4797-b234-2118360117d3&scheme=1)

1 **Utilization of adequately iodized salt and its associated factors in Tanzania rural areas: a case of**
2 **Kilwa district, Lindi region, 2023**
3 David Mahwera^1,2^, Rose Msaki^3^, Rogath Kishimba^2,4^, Fatma Abdallah^3^, Danford Mahwera^5^,
4 Vicent Assay^3^, George Mrema^1,2,4*^, Geofrey Mchau^1,2,3^, Germana Leyna^1,3^ Theresia Ambrose^1^
5 *Email: [drgeorgemrema@gmail.com](mailto:drgeorgemrema@gmail.com) (GM)
6 **Authors Affiliation**
7 ^1^Department of Epidemiology and Biostatistics, Muhimbili University of Health and Allied
8 Sciences, Dar es Salaam, Tanzania
9 ^2^Tanzania Field Epidemiology and Laboratory Training Program, Ministry of Health, Dar es
10 Salaam, Tanzania
11 ^3^Department of Community Health and Nutrition, Tanzania Food and Nutrition Centre, Ministry
12 of Health, Dar es Salaam, Tanzania
13 ^4^Epidemiology and Disease Control Section, Ministry of Health, Dodoma, Tanzania
14 ^5^College of Engineering, University of Dar es Salaam, Dar es Salaam, Tanzania

# 15 Abstract

16 **Background:** Salt with adequate iodine content is crucial for preventing iodine
17 deficiency disorders. However, the utilization of adequately iodized salt in Tanzania regions varies,
18 and the situation is worse in areas with local salt production sites. Hence, this study aimed to assess
19 household-level utilization of adequately iodized salt and its associated factors in Kilwa district,
20 Tanzania.
21 **Methodology:** A cross-sectional research design was used in this study. A total of 493 households
22 were systematically sampled to join the study and one-on-one interviews were conducted with the head
23 of each household. Purposive sampling was used to recruit 14 local salt producers and in-depth
24 interviews were conducted with them. AnIodine rapid test was performed onsite followed by iodine
25 content analysis at Kigoma Regional Referral Hospital (RRH) laboratory. Quantitative data were
26 analyzed using STATA while NVivo software was applied to analyzed the qualitative data.
27 The association between dependent and independent variables was assessed by binary logistic
28 regression.
29 **Result:** Only 9.4% of households utilized adequately iodized salt. Family size with equal or less
30 than five members (Adjusted Odds Ratio (AOR), 3.49; 95% CI: 1.62, 7.54), good knowledge about
31 iodized salt (AOR. 4.97; 95% CI: 2.04, 12.11), salt storage in a dry area (AOR, 4.44; 95% CI: 1.51,
32 13.07), expose salt to sunlight (AOR, 0.29; 95% CI: 0.10, 0.85), and stay with salt for less
33 than two months (AOR, 2.34; 95% CI: 1.10, 5.00) were significantly associated with the utilization of
34 adequately iodized salt. Poor protection of salt at the production sites, supply of non-iodized salt
35 to workers, unavailability of training for local salt producers, presence of other local salt producers
36 in the community, and community preference for non-iodized salt were the reasons for the low
37 availability of iodized salt in Kilwa district as reported by local salt producers.
38 **Conclusion:** The prevalence of utilization of adequately iodized salt in Kilwa district is very low.
39 Family size, knowledge about iodized salt, storage place, exposure to sunlight and, salt stay time
40 were associated with the utilization of adequately iodized salt. Local salt producers contribute a lot to
41 the poor availability of iodized salt. It is recommended among others that the Tanzania Government
42 should put more efforts into awareness campaigns to raise knowledge in the study area on the benefits
43 of using iodized salt.
44 **Keywords:** Iodized salt, Iodine deficiency, Utilization, Rural Tanzania

# 45 Introduction

46 Iodine is a micronutrient that is necessary at modest levels for both human and animal physiologic
47 function. It is a crucial part of thyroid hormones, which are essential for regulating metabolic rate,
48 growth, and development of bodily structures as well as neuronal function and growth [1]. Iodine
49 deficiency and related illnesses are acknowledged as one of the main micronutrient issues of public
50 health concern in Tanzania. Approximately 25% of the population was said to have an iodine
51 deficiency condition, while 41% of people lived in regions that had an iodine shortage [2]. The
52 World Health Organization (WHO) recommends consuming 150 μg/day for adults, 13 g/day for
53 teenagers, 200 g/day for women during pregnancy and breastfeeding, 120 g/day for children 6 to
54 12 years old, and 90 g/day for infants 0 to 59 months old [3]. Nearly 2 billion individuals
55 worldwide are still affected by iodine deficiency illnesses which put them at risk for cognitive
56 impairment, stillbirth, and irreparable brain damage [1,4,5]. Individuals from similar populations
57 in regions without severe iodine shortage may have intelligence **quotients** that are up to 13.5 points
58 **higher** than those in places with severe iodine deficiency [6].
59 The main cause of thyroid pathology in Africa is dietary iodine insufficiency, which leads to a
60 variety of iodine deficiency illnesses, including goiters, hypothyroidism, and mental retardation,
61 the last of which presents the greatest risk to socioeconomic well-being. Iodine deficiency poses a
62 threat to 350 million Africans at the very least. Goiters are estimated to affect 28.3% of Africans,
63 and the continent accounts for around 25% of the world's iodine deficiency burden as measured
64 by disability-adjusted living years (DALYs) according to estimates from the WHO [7]. In
65 Tanzania especially in the Southern Highlands regions, the total goiter prevalence (TGP) was up
66 to 90%, and 50% of schoolchildren had hypothyroidism [8].
67 The global strategy for the eradication of iodine deficiency disorder (IDD) was identified as
68 universal salt iodization (USI), which aims to iodize all salt for human and animal consumption in
69 order to ensure adequate iodine nutrition. This strategy has been proposed in numerous studies
70 carried out in various locations [9].
71 Around the world, 70% of households consume adequate iodized salt, and more than 120 nations
72 are running USI initiatives. Of these, 34 countries that have already reached USI, another 28 are
73 almost there. Additionally, 84 million children are shielded each year from the danger of IDD, and
74 the number of nations where the issue is still present has fallen to 47 [10].
75 USI has been adopted in Tanzania as a means of eliminating IDD in the country. USI goal is to
76 ensure that household coverage in Iodated salt utilization reaches 90%, however, Tanzania still
77 have a coverage of 61.2% as per Tanzania National Nutrition Survey of 2018 [11]. Iodized salt
78 that contain >15ppm and <40 ppm were considered adequately iodized for the prevention of iodine
79 deficiency [6]. Low coverage of iodized salt may place the community a risk of irreversible brain
80 damage, stillbirth, and cognitive impairment [1]. In Tanzania, it was reported that low iodine
81 utilization is mainly in rural areas and might be caused by the low knowledge on the importance use
82 of iodized salt utilization, inadequate availability of iodized salt in the community, price of iodized
83 salt and cultural beliefs in the community (Tanzania Food and Nutrition Centre (TFNC),
84 unpublished annual report of 2021). This study provides the current prevalence of iodized salt
85 utilization at the household level in rural and explore the factors that influence the household level
86 utilization of iodized salt in the community as well as the reasons for inadequate availability of
87 iodized salt in the community among local salt producers in Kilwa, Tanzania. By knowing the
88 current prevalence of households utilizing iodized salts, factors associated with iodized salt
89 utilization and reasons for adequate availability of iodized salt in the community among local salt
90 producers in Kilwa, the government through TFNC will be able to design appropriate intervention
91 to improve the coverage of household and to help local salt producers to produce salt which is
92 adequately iodized.

93

# 94 Materials and methods

## 95 Study design, area and population under the study

96 A cross-sectional study was carried out in April 2023 in the Lindi region. Both quantitative and qualitative
97 methods were utilized for data collection. Lindi region is located in southern part of Tanzania
98 mainland having a population of 864,652 with an estimated growth rate of 0.9% and an area of 15,000
99 km^2^ and, with 225,972 households (24). The region has one municipal council called Lindi
100 municipal council and, five district councils namely Kilwa district council, Lindi district council,
101 Liwale district council, Nachingwea district council and Ruangwa district council. The main
102 source of income in Lindi region is agriculture [12]. Lindi was chosen because it has a lower
103 proportion of households utilizing adequately iodized salt and the presence of local salt production
104 sites Within Lindi region, Kilwa district was purposively chosen because it is the worst performer
105 among all districts in Lindi region (Unpublished TFNC reports).
106 The study involved heads of households and local production sites in charges from
107 Mandawa,Kiranjeranje, Miteja and Tingi wards (fig 1).
108 **Fig 1. Map of Kilwa district, Lindi region, Tanzania, illustrating the study area for assessing**
109 **household utilization of adequately iodized salt and associated factors. The** map was drawn using
110 QGIS desktop software 3.28.3. The shapefiles used were from an openly available source
111 (<https://www.nbs.go.tz/statistics/topic/gis>). The shapefiles were made based on the 2012
112 population and housing census, but in this study, shapefiles have been modified to capture all the
113 regions information.

## 114 Sample size and sampling technique

115 We estimated the minimum sample size of 493 households using a formula for estimating a sample
116 for proportions [13], considering previous proportion of households using adequately iodized salt
117 at Lindi 37.6% [11]. A two-stage cluster sampling design was used in the sample selection. In
118 executing this sampling design, the first stage involved random selection of 4 wards (Mandawa,
119 Kiranjeranje, Miteja, Tingi) from a list of available wards in Kilwa district and the second stage
120 involved systematic selection of 493 households from 4 selected wards. Households were picked
121 in the interval of 6 at Mandawa ward, 5 at Kiranjeranje ward, 3 at Miteja ward and 3 at Tingi ward.
122 The selection interval was obtained by dividing the total households in each selected ward by the
123 desired sample size. The first house was picked randomly by spinning a pen then the following
124 households were picked in north direction.
125 For the qualitative part of the study, a convenience sampling method was utilized to select 14 local
126 production sites out of 101 sites present in Kilwa district. Sites which were accessible and its
127 hamlet leader was present in a day of interview was the one chosen for this study. It has been
128 previously mentioned that at least 12 respondents are sufficient to reach data saturation in
129 qualitative studies [14]. Consequently, a purposive sampling approach was used to recruit 14 locals
130 production sites in charges. A purposive sampling aim to select participants who are more
131 knowledgeable about the problem under investigation and was chosen based on years of
132 experience in salt production [15]**.**

## 133 Data measurement and quality control

134 Data collection was conducted by trained research assistants under the supervision of the principal
135 investigator from April 3 to 29, 2023. Training was provided to all research assistants in the use
136 of questionnaires and other data collection tools. Each data collector was provided with
137 questionnaire in paper based, rapid test kits (RTKs) for testing salt samples on the site and plastic
138 bags for salt sample collection and storage ready for laboratory analysis.
139 Semi Structured and pre-tested questionnaires were used to conduct face to face interviews with
140 the heads of households to collect information on factors associated with adequately iodized salt
141 utilization. However, an interview guide with open ended questions was used to conduct face to face
142 in-depth interview with site in charges to collect information on the profile of the recruited local
143 production sites at Kilwa district. In-depth interviews were audio recorded by a digital audio
144 recorder for transcription, translation and coding.
145 Before starting the process of data collection, data collection tools (questionnaire and in-depth
146 interview guide) were pre-tested among 12 number of head of households and 2 number of local
147 production sites in-charges, in one of the wards namely Masoko ward within Kilwa districts which
148 was not part of wards for this study. Masoko ward has similar characteristics.
149 Salt samples (a tea spoonful) collected from the households and local production sites were
150 qualitatively tested for iodine using iodine rapid testing kit [16]. A salt samples which turned to
151 blue or purple color after being exposed to a drop of test solution were considered to be iodized
152 [17]. All salt samples which were positive for the rapid test were placed into a screwed-capped
153 plastic container and appropriately labeled ready for being transported to Kigoma Regional
154 Referral Hospital (RRH) laboratory to be tested for iodine content. The laboratory analysis of these
155 salt samples were performed by using WYD machine [18].

## 156 Data processing and analysis

157 Quantitative data was collected by using questionnaires in paper form, was then entered in excel
158 for cleaning and then transported to STATA version 15 for analysis. In this study both descriptive
159 and inferential statistics were used to analyze quantitative data. All data are summarized in tables
160 and figures for clear understanding. Bivariable and multivariable logistic regression were used to
161 find the association between the dependent and independent variables. During bivariable analysis,
162 variables with *р* ≤ 0.2 were included in multivariable analysis. To compensate for the potential
163 confounding influence of independent factors, a multivariable analysis was performed. In
164 multivariable analysis, variables with *р* ≤ 0.05 was considered significantly associated with
165 utilization adequately iodized salt [19].
166 Qualitative data was audio recorded. Content analysis approach was used to analyze qualitative
167 data using NVivo software [20]. During the content analysis approach, the researcher first
168 transcribed all recorded information into word document. During this process the researcher
169 ensured that all transcribed data are clean by eliminating all irrelevant information. Thereafter,
170 transcribed data was translated into English language and imported into NVivo software, carefully
171 coded and finally organized into appropriate themes [21].

## 172 Definition of terms

173 **Adequately Iodized Salt:** Salt that contains ≥15 parts of iodine per million parts of salt, assessed
174 by taking salt samples that become positive to RTK on site and sent in laboratory for quantitative
175 analysis [22].
176 **Partially Iodized Salt:** Salt that contains less than 15 parts of iodine per million parts of salt
177 assessed by taking salt samples that become positive to RTK on site and sent in laboratory for
178 quantitative analysis.
179 **Non-Iodized Salt:** Salt that has no iodine which may cause a lot of public health problems such
180 as congenital malformations, stillbirth, brain damage, etc. that was assessed by testing salt sample
181 on site by rapid test kit (RTK).
182 **Utilization of adequately iodized salt:** This is the act of using/uptake or eating salt that contains
183 ≥15 parts of iodine per million parts of salt. This study regarded participants with adequately
184 iodized salt at home as participants who utilize adequately iodized salt.
185 **Household:** As per this study household is a group of individuals who live together and share the
186 same cooking and living space [11].

## 187 Ethical clearance

188 Ethical approval was obtained from the ethical review board at Muhimbili University of Health
189 and Allied Sciences with approval number MUHAS-REC-11-2022-1462. Participants were given
190 explanation on the objectives of the study then written and verbal informed consent was given to
191 the participants; only consented participants were involved in the study. Name and other personal
192 identifiers were excluded in the questionnaire and only identification numbers were used instead
193 to ensure confidentiality. Permission to enter into selected wards was obtained from Ward
194 Executive Officers (WEOs).

195

# 196 Results

## 197 Socio-demographic and economic characteristics

198 Out of the 493 visited households, 435 heads of households were available to participate in the
199 study, resulting in a response rate of 88.2%. The characteristics of these respondents are provided
200 below. Two hundred and seventy-three (62.8%) of the respondents were female. Regarding age,
201 majority 252(57.9%) of the respondents were in the age group of 26 – 35 years. Majority 223
202 (51.3%) of the respondents had primary level of education, and 344 (79.1%) had low level of
203 income (less than Tshs. 100, 000 per month). Peasants contributing to 239(54.9%) and households
204 with more than five family members had 239(54.9%) of the total respondents. Table 1 summarizes
205 the findings.
206 **Table 1. Socio-demographic and Economic Characteristics of the Respondents in Kilwa**
207 **District, Lindi Region (n=435)**

| **Variable** | **Category** | **Frequency (%)** |
| --- | --- | --- |
| Gender | Female | 273 (62.8) |
|  | Male | 162 (37.2) |
| Age Group (Years) | Below 18 yrs. | 16 (3.7) |
|  | 18-25 | 28 (6.4) |
|  | 26-35 | 252 (57.9) |
|  | 36-45 | 74 (17.0) |
|  | Above 45 yrs. | 65 (14.9) |
| Level of Education | Illiterate | 115 (26.4) |
|  | Primary Education | 223 (51.3) |
|  | Secondary Education | 79 (18.2) |
|  | Higher Education | 18 (4.1) |
| Source of income | Government Employees | 13 (3.0) |
|  | Merchants | 52 (12.0) |
|  | Self-employed | 131 (30.1) |
|  | Peasants | 239 (54.9) |
| Income level | Low | 344 (79.1) |
|  | Average | 86 (19.8) |
|  | High | 5 (1.1) |
| Family size | >5 members | 239 (54.9) |
|  | ≤5 members | 196 (45.1) |

## 208 Proportion of Households that Use Adequately Iodized Salt

209 Out of 435 collected salt samples from surveyed households, only 41 (9.4%) households were
210 found utilizing salt with adequate iodine content (≥ 15 ppm) while 394 (90.6%) households had
211 inadequately iodized salt (< 15 ppm) (fig 2).
212 **Fig 1. Level of iodine content in the collected salt samples in households at Kilwa district,**
213 **2023**

## 214 Factors influencing utilization of adequately iodized salt

215 Table 2 shows that of all variables analyzed during binary logistic regression only gender, family
216 size, cultural belief, knowledge about iodized salt, storage place, expose to sunlight, time to stay
217 with salt and perceived price of iodized salt are associated with utilization of adequately iodized
218 salt. However, during multivariable logistic regression, family size, knowledge about iodized salt,
219 storage place, expose to sunlight and time to stay with salt significantly influence utilization of
220 adequately iodized salt.
221 The odds of utilization of adequately iodized salt are 3.49 (AOR, 3.49; 95%CI: 1.62, 7.54) times
222 higher among households with family size with equal or less than five members compared with
223 households with more than five family members. Moreover, the odds of utilization of adequately
224 iodized salt among households with good knowledge about iodized salt are 4.97 (AOR. 4.970;
225 95%CI: 2.04, 12.11) times higher compared with households with poor knowledge. Additionally,
226 households which store their salt in dry area are 4.44 (AOR, 4.44; 95% CI: 1.51, 13.07) times more
227 likely to utilize adequately iodized salt than households which store their salt near fire. Again, the
228 odds of utilization of adequately iodized salt are decreased by 70% (AOR, 0.30; 95%CI: 0.10,
229 0.85) among households which expose their salt at sunlight compared to non-expose to sunlight.
230 Lastly, households which stay with salt for less than two months are 2.34 (AOR, 2.34; 95% CI:
231 1.10, 5.00) times more likely to utilize adequately iodized salt than households which stay with
232 salt for more than two months.
233 **Table 2. Factors associated with adequate iodized salt utilization among households in Kilwa**
234 **district, 2023 (n =435)**

| **Variables** | **Total** | **Yes** | **Crude Odds Ratio (95% CI)** | **Adjusted Odds Ratio (95% CI)** |
| --- | --- | --- | --- | --- |
| **Gender** |  |  |  |  |
| Female | 273 | 22 (8.1%) | 1 |  |
| Male | 162 | 19 (11.7%) | 1.52 (0.79, 2.90) | 1.81 (0.84, 3.94) |
| **Age** |  |  |  |  |
| Below 18 | 16 | 1 (6.3%) | 1 |  |
| 18-25 | 28 | 2 (7.1%) | 1.15 (0.10, 13.82) |  |
| 26-35 | 252 | 28 (11.1%) | 1.88 (0.24, 14.74) |  |
| 36-45 | 74 | 4 (5.4%) | 0.86 (0.09. 8.22) |  |
| Above 45 | 65 | 6 (9.2%) | 1.53 (0.17, 13.65) |  |
| **Level of** |  |  |  |  |
| **Education**  Illiterate | 115 | 10 (8.7%) | 1 |  |
| Primary education | 223 | 21 (9.4%) | 1.09 (0.50, 2.40) |  |
| Secondary | 79 | 7 (8.9%) | 1.02 (0.37. 2.81) |  |
| education |  |  |  |  |
| Higher education | 18 | 3 (16.7%) | 2.10(0.52, 8.51) |  |
| **Family size** |  |  |  |  |
| >5 members | 239 | 12 (5.0%) | 1 |  |
| ≤5 members | 196 | 29 (14.8%) | 3.29 (1.63, 6.63) | 3.49(1.62, 7.54) ** |
| **Income source** |  |  |  |  |
| Government | 40 | 6 (15.4%) | 1 |  |
| Merchants | 209 | 19 (13.5%) | 0.86 (0.16, 4.70) |  |
| Self-employed | 130 | 10 (8.4%) | 0.50 (0.10, 2.57) |  |
| Peasants | 56 | 6 (8.8%) | 0.53 (0.11, 2.55) |  |
| **Income level** |  |  |  |  |
| Low | 344 | 30 (8.7%) | 1 |  |
| Average | 86 | 10 (11.6%) | 1.38 (0.65, 2.94) |  |
| High | 5 | 1 (20.0%) | 2.62 (0.28, 24.17) |  |
| **Salt preference** |  |  |  |  |
| Common salt | 278 | 28 (10.1%) | 1 |  |
| Rock salt | 157 | 13 (8.3%) | 0.81 (0.41, 1.61) |  |
| **Cultural beliefs** |  |  |  |  |
| Yes | 216 | 14 (6.5%) | 1 |  |
| No | 219 | 27 (12.3%) | 2.03(1.03, 3.99) | 1.89 (0.87, 4.10) |

| **Variables** | **Total** | **Yes** | **Crude Odds Ratio (95% CI)** | **Adjusted Odds Ratio (95% CI)** |  |
| --- | --- | --- | --- | --- | --- |
| **Knowledge about iodized salt** |  |  |  |  |  |
| Poor | 215 | 7 (3.3%) | 1 |  |  |
| Good | 220 | 34 (5.5%) | 5.43 (2.35, 12.55) | 4.97 (2.04, 12.11) * |  |
| **Storage place** |  |  |  |  |  |
| Near fire | 111 | 5 (4.5%) | 1 |  |  |
| Moist area | 189 | 10 (5.3%) | 1.18 (0.39, 3.56) | 1.19 (0.37, 3.82) |  |
| Dry area | 135 | 26 (19.3%) | 5.06 (1.87, 13.66) | 4.44 (1.51, 13.07) ** |  |
| **Type of storage container** |  |  |  |  |  |
| Container with lid | 50 | 7 (14.0%) | 1 |  |  |
| Plastic bag | 303 | 27(8.9%) | 0.60 (0.45, 1.47) |  |  |
| Container without lid | 82 | 7 (8.5%) | 0.57 (0.19, 1.74) |  |  |
| **Expose to sunlight** |  |  |  |  |  |
| No | 318 | 36 (11.3%) | 1 |  |  |
| Yes | 117 | 5 (4.3%) | 0.35 (0.13, 0.91) | 0.30 (0.10, 0.85) ** |  |
| **Duration you stay with salt** |  |  |  |  |  |
| > 2 months | 262 | 14 (5.3%) | 1 |  |  |
| ≤ 2 months | 173 | 27 (15.6%) | 3.28 (1.66, 6.45) | 2.34 (1.10, 5.00) * |  |
| **Salt buying place** |  |  |  |  |  |
| Open Market | 181 | 17 (9.4%) | 1 |  |  |
| Salt farms | 88 | 9 (10.2%) | 1.10 (0.47, 2.58) |  |  |
| Local shops | 166 | 15 (9.0%) | 0.96 (0.46, 1.99) |  |  |
| **Distance to get salt**  ≤30 minutes | 368 | 34 (9.2%) | 1 |  |  |
| >30 minutes | 67 | 7 (10.4%) | 1.15 (0.49, 2.71) |  |  |
| **Perceived cost of iodized salt** |  |  |  |  |  |
| Cheap | 49 | 8 (16.3%) | 1 |  |  |
| Expensive | 386 | 33 (8.5%) | 0.48 (0.21, 1.11) | 0.82 (0.31, 2.47) |  |

235 *P-value<0.05, **p-value<0.01, ***p-value<0.001 Significant on multivariable logistic regression

## 236 Reasons for Inadequate Availability of Adequately Iodized Salt

### 237 Poor protection of salt production sites

238 Low availability of adequately iodized salt in the study area was also caused by poor protection of
239 salt production sites as one of the respondents mentioned that:
240 *…after collecting it in the fields because there are no guards, people go there and steal as*
241 *much as they see fit* (Respondent 2).
242 Similar to this opinion, another respondent argued that:
243 *…the salt without iodine is often stolen and people take it to the street to sell* (Respondent
244 3).

### 245 Supply of non-iodized salt to workers

246 Findings from interview also revealed that a tendency of in charges of local production sites to
247 provide non-iodized salt to their workers free of charge contribute to poor utilization of adequately
248 iodized salt in Kilwa district. This was revealed by the majority of the participants. One of the
249 respondents had the following to say during interview session:
250 *…No, we don’t give salt as wages, but when we finish harvesting, they are given salt for*
251 *use in their homes* (Respondent 4).
252 Similar view was given by another respondent who asserted:
253 *…Yes, sometimes when there is not enough money, we usually give them about two sacks*
254 *of salt to sell and earn money, we don't put iodine in salt until we are transporting it, so*
255 *they get salt without iodine* (Respondent 5).

### 256 Unavailability of training to local salt producers

257 Absence of formal training to local salt producer was another reason for poor availability of
258 adequately iodized salt in the study area as stated by the majority of the participants. During
259 interview session one the respondent had the following to say:
260 *For about 3 to 4 years there has been no training in salt production or handling*”
261 (Respondent 1).
262 Similarly, another respondent highlighted that:
263 *…There is no special training, but we teach each other in the field. We have never been*
264 *given formal education from the district level or other lower levels*” (Respondent 7).

### 265 Presence of other local salt producers in the community

266 It was found that in the community there some people who are producing salt at their home in a
267 small amount which was not enough to be transported outside the village and hence they just
268 produce for selling in the local market and using at home
269 *“… some people cook salt in their homes for their own use and sometimes they sell it to*
270 *people in the village”* (Respondent 2*).*

### 271 Community preference on non-iodized salt

272 The study also reviled that the majority in the community including the local salt producers
273 want/prefers salt that is straight coming from the farm which is not added with anything including
274 iodine(non-iodized)
275 “*However, people don't want industrial salt, they demand it contains chemicals, that's why*
276 *they fail to reproduce, people believe that salt like salt from the sea is good and it gives you*
277 *strength”* (Respondent 2*).*
278 Similarly, another respondent highlighted that:
279 “... *although the community and homes do not want salt with iodine”* (Respondent 1).

# 280 Discussion

281 Findings of this study revealed that 9.4% of households in Kilwa district utilize adequately iodized
282 salt (≥ 15ppm) which is lower than 90% recommended by WHO. Based on these findings, one
283 may argue that despite of existence of universal salt iodization programme in the country a large
284 number of households in Kilwa district are at high risk of developing iodine deficiency illnesses.
285 Low availability of adequately iodized salt in the study area might be attributed to households’
286 poor salt storage practice and/or poor technology employed by local salt producers. The proportion
287 of households with adequately iodized salt found in this study is lower compared to studies
288 conducted in Tach Armachio and Ahferom districts in Northern Ethiopia which were 61.1% and
289 17.5%, respectively [23,24]. Additionally, this value is also lower than 75% in a study conducted
290 in Prakasam district, India [25]. Moreover, the observed prevalence of utilization of adequately
291 iodized salt in the study area is still lower than recently reported values in two Himalayan districts;
292 89% in Una district and 91% in Hamirpur district [26]. Due to low utilization of adequately iodized
293 salt contributes to iodine deficiency disorders (IDD), including goiter problems [2]. To address
294 this problem, it is advised to Increase awareness and education on the importance of adequately
295 iodized salt utilization, ensure the consistent availability and accessibility of adequately iodized
296 salt in the community by Working with local salt producers, suppliers, and retailers to ensure a
297 reliable supply chain that can distribute iodized salt to households and local markets. By
298 implementing these solutions, it is possible to increase the utilization of adequately iodized salt,
299 reduce the prevalence of iodine deficiency disorders, and improve the overall health and well-
300 being of the community.
301 Findings of this study indicated that, household with equal or less than five members was
302 positively associated with utilization of adequately iodized salt compared to households with more
303 than five family members. This is possibly due to the high price of iodized salt at the study area,
304 hence, as a family becomes large, it opts to purchase cheaper salt which is mostly non-iodized.
305 This finding aligns with a study done in Dire Dawa City in Ethiopia which reported that utilization
306 adequately iodized salt was reduced to 61% for households with more than five members [27].
307 Education on the importance of using adequately iodized salt is very key to the change toward
308 utilization and attaining USI goal, supporting local producers will Encourage local production of
309 iodized salt, either through support for small-scale salt producers or by establishing community-
310 based iodization programs. This can help increase the availability of adequately iodized salt at a
311 more affordable price, benefiting households with larger family sizes.
312 Moreover, the odds of utilization of adequately iodized salt among households with good
313 knowledge about iodized salt are 4.97 times higher compared with households with poor
314 knowledge. This might be due to the fact that households with good knowledge regarding the effect
315 of non-use of adequately iodized salt help them to make good decision when purchasing salt.
316 The finding of this study is consistence with those reported in Gidami district, Ethiopia which indicated
317 that the utilization of adequately iodized salt was higher among households with good knowledge
318 on iodized salt [28].
319 This study revealed that, the odds for utilization of adequately iodized salt among households
320 storing their salt in dry areas are higher compared to those storing their salt near fire. This finding
321 concurs with a study done in Dera district in Ethiopia whereby the ability of salt stored in dry areas
322 to retain iodine content was higher than salt stored near fire areas [29]. Findings of this study also
323 indicated that, utilization of adequately iodized salt were 70% lesser among households exposed
324 their salt to sunlight compared to those who did not. Again, this finding aligns with a study done
325 in Tach Armachio district in Northern Ethiopia which reported that utilization of salt with adequate
326 iodine content was reduced to 61% for households exposed their salt to sunlight [23]. It has been
327 mentioned that salt tends to lose iodine when stored in areas such as heat, moisture and light during
328 storage [30].
329 Lastly, time to stay with salt was also found significantly associated with utilization of adequately
330 iodized salt. It was revealed that, as household stay with salt for less than two months, the chance
331 of utilizing adequately iodized salt increase. Findings of this study is consistence with a study
332 carried out in Dera district, Ethiopia in which the availability of adequate iodine content was more
333 likely to be found in salt samples stored for less than two months compared to salt samples stored
334 for more than two months [29].
335 This study revealed that poor protection of salt production sites was among the reason that hindered
336 the availability of adequately iodized salt in the community. This study identified and observe the
337 presence of salt sites without being protected as a results salt production sites are vulnerable to
338 unauthorized access and theft. Individuals or groups may exploit the lack of security to steal salt
339 which is not iodized and take home for their use or to sell at their local market leading to a shortage
340 in the supply of adequately iodized salt to the community. This was contrary to the study conducted
341 in Madagascar that require the production sites to be registered before commencing the production,
342 the study revealed that before a place of production is registered or given license to function, it
343 must be examined using a certification checklist to guarantee that the "must have items” essential
344 for the manufacture of adequately iodized salt are present. The problem of production site
345 protection was addressed in the check list by assuring the existence of "fence or wall and clean
346 from rubbish and debris, animals, and unauthorized person passage are assessed. The study in
347 Madagascar revealed that their salt production site was well protected [31]. Poor protection of salt
348 production sites in Kilwa district might be attributed to lack of awareness among local salt
349 producers as local producers may not fully understand the importance of protecting salt production
350 sites and the implications of inadequate security measures. They may not be aware of the potential
351 risks, such as theft, contamination, or adulteration, and how these can affect the availability of
352 adequately iodized salt. Also, it might be due to limited resources as local salt producers, especially
353 small-scale ones, may have limited financial resources to invest in security measures and lastly, it
354 might be due to the weak enforcement and regulation as there might be a lack of strict enforcement
355 and regulation by local authorities or government agencies regarding the protection of salt
356 production sites.
357 Additionally, it was found that workers were given salt to use in their homes, but in some sites,
358 the amount provided was intended for them to sell in the market. The results from this study
359 contradict with Nepal iodized salt act which prohibit the importation or distribute iodine free salt
360 to the community [32]. This practice can be attributed to the owners or site supervisors attempting
361 to compensate the workers by providing salt as a form of wages that they can sell to earn money.
362 This situation typically arises when there is a shortage of customers to purchase the harvested salt,
363 resulting in a lack of funds to pay the workers for their services. To address this issue, the owners
364 or site supervisors opt to give bags of salt to the workers to sell on their behalf. However, since
365 iodine is typically added during transportation or when customers are present, the salt provided for
366 sale is often non-iodized. This practice not only compromises the availability of adequately iodized
367 salt in the market but also poses a risk to the workers who rely on the income generated from
368 selling the salt. It perpetuates the cycle of non-iodized salt consumption and contributes to the
369 overall low utilization of adequately iodized salt in the community.
370 Moreover, this study found that there is unavailability of training to local salt producers in Kilwa
371 district. These findings were in line with study another conducted in Tanzania which also indicated
372 that unavailability of training caused production of inequality salt a situation which was improved
373 by giving training to local salt producers [33]. The training helps the local salt producers to know
374 the importance of adding iodine to the salt, proper storage as well as the right concentration to add.
375 The lack of training in Kilwa might be attributed by the absence of coordination and collaboration
376 among different stakeholders involved in salt production and iodization.
377 It is anticipated that all salt producers in the community were supposed to be recognized by the
378 authority (Kilwa District Health Management Team) but this study revealed the presence of other
379 local salt producers in the community. These were the people who were producing salt at their
380 home place and were not recognized by the authority. It was found that they were producing the
381 amount that was insufficient to be transported outside the district but enough to be taken to their
382 local markets. These producers don’t add Iodine to the salt they produce as a result they tend to
383 increase the problem of inadequate availability of adequately iodized salt in the community. The
384 presence of these local salt producers might be caused by the difficulty in life as a result they try
385 to produce salt for use in their homes and generating income for their families by selling the salt
386 they produce. Despite of hardships of life also lack of knowledge about iodized salt led to these
387 people to produce and sell salt without adding iodine.
388 Lastly, the study revealed that the community prefers the use of non-iodized salt over iodized salt.
389 This was another reason given by local salt producers to why there is non-iodized salt in the
390 community, they say people in the community (Kilwa district) including themselves prefer to use
391 salt that is directly coming from the farm they believe it is not added with anything and it is pure.
392 This is contrary to the study conducted in Dessie and Combolcha Town, South Wollo, Ethiopia,
393 and the study was done in Prakesam district in India which revealed that the majority (80.4%) and (83.6%) of
394 participants respectively prefer iodized salt over non iodized salt [16,25]. The preference of non-
395 iodized salt over iodized salt might be due to the presence of cultural believes in the community
396 that they believe iodized salt is not good for their health and might cause sterility to both men and
397 women. Due to this, local producer has no other options other than supplying non-iodized salt to
398 meet the demand. Other reasons might be due to poor knowledge about iodized salt among both
399 local salt producers and community as a result they don’t know the benefits and consequences of
400 supplying and using non-iodized salt in their community.
401 This study faced the limitation of refusal from some participants to participate in the study, this
402 limitation may result in response bias, and may cause the distortion of results as it might result to
403 underestimation of the prevalence of people utilizing adequately iodized salt since the people who
404 refused might have the outcome of interest.
405 To mitigate this, adjustment for Non response was considered to reduce the effect and also the
406 persuasion of the people was done by the help of hamlet leaders by clearly explaining the
407 objectives and the benefits he/she will get by participating in this study.
408 Despite the limitation faced, this study has results that will add to researchers in gaining more
409 knowledge on utilization of adequately iodized salts and its associated factors in rural areas
410 especially areas with Local salt producer.

# 411 Conclusion

412 The prevalence of utilization of adequately iodized salt in Kilwa district is very low. Family size,
413 knowledge about iodized salt, storage place, exposure to sunlight and salt stay time were associated
414 with utilization of adequately iodized salt. Local salt producers contribute a lot to poor availability
415 of iodized salt. It is recommended among others that Tanzania Government should put more efforts
416 on awareness campaigns to raise knowledge in the study area on benefits of using iodized salt.

# 417 Acknowledgement

418 We would want to thank everyone who took the time to ensure that this task was completed. I
419 appreciate the heads of households from Kilwa district who participated in this study and the Ward
420 Executive Officers from five wards for their assistance during data collection. I’m also grateful to
421 Mr. Adolf Kalombo and Joseph Kimambo for assisting me in collecting the data at the field.

# 422 Author Contributions

423 **Conceptualization:** David Mahwera, Rose Msaki, Rogath Kishimba, Fatma Abdallah, Vicent
424 Assay, Geofrey Mchau, Germana Leyna, Theresia Ambrose
425 **Data curation:** David Mahwera, Geofrey Mchau
426 **Formal Analysis:** David Mahwera, Rose Msaki and Geofrey Mchau, Theresia Ambroce
427 **Investigation:** David Mahwera
428 **Methodology:** David Mahwera, Fatma Abdallah, Danford Mahwera, George Mrema, Geofrey
429 Mchau, Theresia Ambrose
430 **Supervision:** Rogath Kishimba, Germana Leyna, Geofrey Mchau, Theresia Ambrose
431 **Validation:** David Mahwera, Rose Msaki, Rogath Kishimba, Fatma Abdallah, Danford Mahwera,
432 Vicent Assay, George Mrema, Geofrey Mchau, Germana Leyna, Theresia Ambrose
433 **Visualization:** David Mahwera, Rose Msaki, Rogath Kishimba, Fatma Abdallah, Danford
434 Mahwera, Vicent Assay, George Mrema, Geofrey Mchau, Germana Leyna, Theresia Ambrose
435 **Writing -Original draft**: David Mahwera, Rose Msaki, Rogath Kishimba, Fatma Abdallah,
436 Danford Mahwera, Vicent Assay, George Mrema, Geofrey Mchau, Germana Leyna, Theresia
437 Ambrose
438 **Writing -review & editing:** David Mahwera, Rose Msaki, Rogath Kishimba, Fatma Abdallah,
439 Danford Mahwera, Vicent Assay, George Mrema, Geofrey Mchau, Germana Leyna, Theresia
440 Ambrose

# 441 References

442 1.	Menyanu E, Corso B, Minicuci N, Rocco I, Zandberg L, Baumgartner J, et al. Salt-
443 reduction strategies may compromise salt iodization programs: Learnings from South
444 Africa and Ghana. Nutrition [Internet]. 2021;84:111065. Available from:
445 https://doi.org/10.1016/j.nut.2020.111065
446 2.	Assey VD, Peterson S, Kimboka S, Ngemera D, Mgoba C, Ruhiye DM, et al. Tanzania
447 national survey on iodine deficiency: Impact after twelve years of salt iodation. BMC
448 Public Health. 2009;9:1–11.
449 3.	Andersson M, Takkouche B, Egli I, De Benoist B. The WHO Global Database on iodine
450 deficiency disorders: The importance of monitoring iodine nutrition. Scand J Nutr.
451 2003;47(4):162–6.
452 4.	Pandav CS, Yadav K, Srivastava R, Pandav R, Karmarkar MG. Iodine deficiency
453 disorders (IDD) control in India. Indian J Med Res. 2013;138(SEP):418–33.
454 5.	Sun D, Codling K, Chang S, Zhang S, Shen H, Su X, et al. Eliminating iodine deficiency
455 in China: Achievements, challenges and global implications. Nutrients. 2017;9(4):1–21.
456 6.	WHO,UNICEF,ICCIDD. Assessment of iodine deficiency disorders and monitoring their
457 elimination. Clin Nutr. 2014;28(2):1–108.
458 7.	Okosieme OE. Impact of iodination on thyroid pathology in Africa. J R Soc Med.

459	2006;99(8):396–401.

460 8.	Wachter W, Mvungi M, Konig A, Pickardt CR, Scriba PC. Prevalence of goitre and
461 hypothyroidism in Southern Tanzania: Effect of iodised oil on thyroid hormone
462 deficiency. J Epidemiol Community Health. 1986;40(1):86–91.
463 9.	Hetzel BS. Eliminating iodine deficiency disorders - The role of the international council
464 in the global partnership. Bull World Health Organ. 2002;80(5):410–3.
465 10.	Mannar MGV, Bohac L. Achieving Universal salt iodization: lesson learned and emerging

466	issues. 2008;(May 2008):1–18.

467 11.	United Republic of Tanzania. Tanzania National Nutrition Survey 2018. 2019. 144 p.
468 12.	the United Republic of Tanzania. LINDI REGION INVESTMENT GUIDE. 2019;
469 13.	Israel GD. Determining Sample Size 1. 1992. 1–5 p.
470 14.	Konstantina Vasileiou, Julie Barnett, Susan Thorpe, Terry Young. Characterising and
471 justifying sample size sufficiency in interview-based studies: systematic analysis of
472 qualitative health research over a 15-year period. BMC Med Res Methodol. 2018;18(1):1–
473 18.
474 15.	Gill SL. Qualitative Sampling Methods. J Hum Lact. 2020;36(4):579–81.
475 16.	Mekonnen TC, Eshete S, Wasihun Y, Arefaynie M, Cherie N. Availability of adequately
476 iodized salt at household level in Dessie and Combolcha Towns, South Wollo, Ethiopia.
477 BMC Public Health. 2018;18(1):1–9.
478 17.	Al-Dakheel MH, Haridi HK, Al-Bashir BM, Al-Shangiti AM, Al-Shehri SN, Hussein I.
479 Assessment of household use of iodized salt and adequacy of salt iodization: A cross-
480 sectional National Study in Saudi Arabia. Nutr J. 2018;17(1):1–7.
481 18.	Carmelle MA, Victoire A, Achille Y, Boris S, Florence A, Roger GJ, et al. Validation of
482 the use of spectrophotometer (WYD iodine checker) for the determination of iodine in
483 food salt. African J Food Sci. 2018;12(2):15–20.
484 19.	Yaye AB, Baraki N, Endale BS. Availability of Adequately Iodized Salt at Household
485 Level and Associated Factors in Dire Dawa, Eastern Ethiopia. Int J Public Heal Sci.
486 2016;5(4):392.
487 20.	Fiona Willtsher. Qualitative Social Research. Forum Qual Sozialforsch / Forum Qual Soc
488 Res [Internet]. 2011;12, No 1(2):345–57. Available from: http://www.qualitative-
489 research.net/index.php/fqs/article/view/865/1880&q=nvivo+manual&sa=x&ei=zah_t5pqo
490 yubhqfe9swgbq&ved=0cc4qfjaj%5Cnhttp://www.qualitative-
491 research.net/index.php/fqs/article/view/1628/3146
492 21.	Hamed Hilal A, Said Alabri S. Using NVivo for data analysis in qualitative research. Int

493	Interdiscip J Educ. 2013;2(2):181–6.

494 22.	Tariku WB, Mazengia AL. Knowledge and Utilization of Iodized Salt and Its Associated
495 Factors at Household Level in Mecha District, Northwest Ethiopia. J Nutr Metab.
496 2019;2019.
497 23.	Mamo W, Derso T, Nigatu SG. Adequately Iodized Salt Utilization and Associated
498 Factors among Households in Tach Armachio District , Northwest Ethiopia : A
499 Community-Based Cross-Sectional Study. 2021;2021:11–4.
500 24.	Desta AA, Kulkarni U, Abraha K, Worku S, Sahle BW. Iodine level concentration,
501 coverage of adequately iodized salt consumption and factors affecting proper iodized salt
502 utilization among households in North Ethiopia: A community based cross sectional
503 study. BMC Nutr. 2019;5(1):1–10.
504 25.	Sai Deepika P, Thirumala Rao B, Vamsi A, Valleswary K, Chandra Sekhar M. A cross
505 sectional study on proper use of iodized salt in communities of rural areas and its relevant
506 factors in Prakasam district, Andhra Pradesh, India. Int J Community Med Public Heal.
507 2019;6(3):1083.
508 26.	Bhardwaj AK, Kumar S, Singh M, Sood A. Household coverage with adequately iodized
509 salt and practices affecting iodine content of salt at household level in two sub- Himalayan
510 districts. 2022;03(04).
511 27.	Kebebew E, Derese T, Bogale G, Berhane A. Iodine concentration level , availability of
512 adequately iodised salt and proper utilisation , and its influencing factors among
513 households in Eastern Ethiopia : a community- - based cross- - sectional study. 2023;
514 28.	Dinka AW, Kebebe T, Nega G. Iodine Level of Salt and Associated Factors at Household
515 Level in Gidami District, Oromia Region, Ethiopia: A Cross-Sectional Study. Nutr Diet
516 Suppl. 2021;Volume 13:9–16.
517 29.	Anteneh ZA, Engidayehu M, Abeje G. Iodine content of dietary salt at household level
518 and associated factors using Iodometric titration methods in Dera District, Northwest
519 Ethiopia. BMC Nutr. 2017;3(1):1–7.
520 30.	Abera T, Deybasso HA, Gebaba EM. Utilization of Iodized Salt and Associated Factors
521 Among Pregnant Mothers in Kimbibit District , North Shoa Zone , Oromia Regional State
522 , Ethiopia. 2021;9(3):89–99.
523 31.	UNICEF. Problems and solutions to improve the Madagascar Iodized Salt Programme |
524 UNICEF Madagascar. 2020;1–42. Available from:
525 https:/[/www.unicef.org/madagascar/en/reports/problems-and-solutions-improve-](http://www.unicef.org/madagascar/en/reports/problems-and-solutions-improve-)
526 madagascar-iodized-salt-programme
527 32.	Democratic Republic of Nepal. Iodized Salt ( Production , Sale and Distribution ) Act.

528	2010;2055(1998):1–17.

529	33.	Assey VD, Tylleskär T, Momburi PB, Maganga M, Mlingi N V., Reilly M, et al.

530	Improved salt iodation methods for small-scale salt producers in low-resource settings in

531	Tanzania. BMC Public Health. 2009;9:1–10.

532

Fig 1	[Click here to access/download;Figure;Fig 1.tif](https://www2.cloud.editorialmanager.com/pone/download.aspx?id=38959739&guid=cc964cc8-9cc3-42eb-94be-e45e0d5163d8&scheme=1)[<https://www2.cloud.editorialmanager.com/pone/download.aspx?id=38959739&guid=cc964cc8-9cc3-42eb-94be-e45e0d5163d8&scheme=1>](https://www2.cloud.editorialmanager.com/pone/download.aspx?id=38959739&guid=cc964cc8-9cc3-42eb-94be-e45e0d5163d8&scheme=1)

Fig 2	[Click here to access/download;Figure;Fig 2.tif](https://www2.cloud.editorialmanager.com/pone/download.aspx?id=38959740&guid=fa5821ab-a62d-49ff-8279-06290c752153&scheme=1)[<https://www2.cloud.editorialmanager.com/pone/download.aspx?id=38959740&guid=fa5821ab-a62d-49ff-8279-06290c752153&scheme=1>](https://www2.cloud.editorialmanager.com/pone/download.aspx?id=38959740&guid=fa5821ab-a62d-49ff-8279-06290c752153&scheme=1)

S1_Data

[Click here to access/download](https://www2.cloud.editorialmanager.com/pone/download.aspx?id=38960688&guid=b7af9f29-3ea5-49ce-9f02-8406adf863f3&scheme=1)

[**Supporting Information** S1_Data.xlsx](https://www2.cloud.editorialmanager.com/pone/download.aspx?id=38960688&guid=b7af9f29-3ea5-49ce-9f02-8406adf863f3&scheme=1)
