# Supplementary material for: Utilization of adequately iodized salt and its associated factors in Tanzania rural areas: a case of Kilwa district, Lindi region, 2023
Source: PLoS One. 2025 Nov 24;20(11):e0337337. doi: 10.1371/journal.pone.0337337 (PMC12643270; doi:10.1371/journal.pone.0337337)
Supplement: Attachment — Submitted filename: PONE-D-25-02467.docx [file pone.0337337.s004.docx]

**Utilization of adequately iodized salt and its associated factors in Tanzania rural areas: a case of Kilwa district, Lindi region, 2023**

1. What is the actual message is not clearly identified?
2. Methods section is very weak.
3. What is the meaning of WYD machine?
4. In 201 no line Mean or median could be included.
5. In table 1, in which basis income level is classified?
6. In 209 line, In laboratory survey, it was found only 91 sample was collected for idodine analysis. Why it is 435? Please justify.
7. From 221-232, Recommendation should be on this ground.
8. In Table 2, In excel sheet data entries 477, but the sample size is 435. what is the reason
9. In Table 2, What were their results on Income source, Income level, Salt preference.
10. In Table 2, How can you explore the cultural beliefs?
11. In Table 2, All this variable should describe in data collection instrument development section. In which basis the categorize the knowledge?
12. In 411 line, re-write with focus on study finding. Recommendation is not match with study finding.
