# Supplementary material for: Utilization of adequately iodized salt and its associated factors in Tanzania rural areas: a case of Kilwa district, Lindi region, 2023
Source: PLoS One. 2025 Nov 24;20(11):e0337337. doi: 10.1371/journal.pone.0337337 (PMC12643270; doi:10.1371/journal.pone.0337337)
Supplement: Attachment — Submitted filename: Response to reviewers comments.docx [file pone.0337337.s006.docx]

**Dear Editor,**

We express our gratitude to the editor and reviewers for their valuable comments, which have significantly enhanced our manuscript titled **"** **Utilization of adequately iodized salt and its associated factors in Tanzania rural areas: a case of Kilwa district, Lindi region, 2023".** We have considered all the comments and integrated their suggestions into the revised version of the manuscript. We believe that we have effectively addressed the raised concerns and that, after incorporating the changes, our manuscript is now appropriate for publication.

Kind regards,

**Dr. George Mrema**

**Reviewer No. 1**

**Comments/concerns/suggestions by reviewer 1:**

1. Language and Typographical Errors: Correct grammatical issues such as (e.g., "expose of salt" → "exposure of salt"; "AnIodine rapid test" → "An iodine rapid test").

**Response to the reviewer:** We appreciate the reviewer's comments. We have corrected all typographical error in the whole manuscript.

2. The introduction ends with a detailed explanation of the study's purpose, but it could be more concise. For example, the sentence "By knowing the current prevalence of households utilizing iodized salts, factors associated with iodized salt utilization and reasons for adequate availability of iodized salt in the community among local salt producers in Kilwa, the government through TFNC will be able to design appropriate intervention to improve the coverage of household and to help local salt producers to produce salt which is adequately iodized" is quite long and could be broken into smaller, more digestible sentences. In addition, the introduction provides good background information, but it should more clearly outline the research gap

**Response to the reviewer:** Thank you so much for the comment. We have revised the last part of our introduction by breaking down the long sentence into smaller, clearer parts and emphasizing the research gap (line 108-117).

3. Provide more detail on the iodine testing methodology

**Response to the reviewer:** We thank the reviewer for pointing out this. The description of Iodine testing methodology is well narrated in methodology part (line 222-230).

4. The discussion jumps between different findings and comparisons without a clear transition. For instance, the section on family size (lines 301–311) is followed by a discussion on knowledge (lines 312–318), then storage practices (lines 319–328), and then time to stay with salt (lines 329–334). While these are all relevant, the flow could be improved by grouping related findings together.

**Response to the reviewer:** We appreciate the reviewer's suggestion. We have rephrased our discussion to improve flow and comparisons our findings and other studies.

**Reviewer No. 2**

**Comments/concerns/suggestions by reviewer 2:**

1. Abstract: Your abstract should adhere to the journal's format, presented as a single continuous paragraph while incorporating all necessary components

**Response to the reviewer:** We appreciate the suggestion. We have changed our abstract into single continuous and adhered into journal standards (line 16-32).

2. Introduction: Please ensure consistency in the WHO iodine intake recommendation unit—whether it is in micrograms or grams. Kindly review and revise the reference statement accordingly in lines 51-54 and throughout the manuscript.

**Response to the reviewer:** Thank you so much for the comment. We have revised and put a new reference in the manuscript (line 69-71).

3. Study Design & Justification: Why was a cross-sectional design chosen for this study, and how does it adequately address the study objectives

**Response to the reviewer:** This design is useful for establishing preliminary evidence for further advanced studies. Moreover, this design is considered inexpensive and easy to conduct. Both quantitative and qualitative methods were utilized for data collection. The use of both quantitative and qualitative methods has a power to generate a clear picture of the problem under study than a single method can yield because weaknesses of one method can be compensated by another method. (Line131-135)

4. Study Design & Justification: Were any potential biases considered in selecting the Kilwa district as the study area

**Response to the reviewer:** Kilwa District was purposively selected for this study based on specific criteria. According to routine quarterly reports from the Tanzania Food and Nutrition Centre (TFNC), Kilwa was identified as the worst-performing district in terms of iodine utilization, making it a suitable site for this investigation.

5. Sampling Methodology: How was the sample size of 493 households determined? Were design effects or response rates considered

**Response to the reviewer:** Thank you so much for the comment. The number of households included in this study was determined using a formula for estimating a sample size for proportions, with adjustments made for a 10% non-response rate and a design effect of 1.226 (Line 154-158).

6. Sampling Methodology: Can the authors provide more justification for the choice of systematic sampling and how it ensures representativeness.

**Response to the reviewer:** Systematic sampling, a probability sampling technique, involves selecting every kth element from a list after a random starting point. It was ideal for our study due to the large population and availability of a comprehensive sampling frame. This method is simple, time-efficient, and minimizes human error. It ensures even distribution across the population, reducing clustering, and helps prevent selection bias through its structured, random approach. To ensure representativeness, the sampling frame was complete, the sample size was sufficient to capture population variability, and the interval (k) was appropriately calculated to ensure proportional representation throughout the population. We have added this in our methodology to increase clarity (Line 161-167)

7. Sampling Methodology: What steps were taken to minimize selection bias, particularly for the qualitative component.

**Response to the reviewer:** To minimize selection bias in the qualitative component, several steps were taken. First, purposive sampling with clear, pre-defined inclusion criteria such as participants’ roles, experience, or exposure to the intervention was employed to ensure the selection of individuals whose perspectives were most relevant to the study objectives. Second, recruitment was conducted across multiple geographic sites to reduce the risk of location-based bias and to capture a broader range of experiences. Lastly, data collection continued until thematic saturation was achieved, ensuring that the analysis reflected a comprehensive range of views and was not disproportionately influenced by a limited subset of participants (Line 177-188).

8. Data Collection & Quality Control: Were there any challenges encountered during data collection, and how were they mitigated

**Response to the reviewer:** During data collection, the main challenges encountered were the unavailability of the head of household at home during the day and language barriers. These challenges were mitigated by involving hamlet leaders, who assisted with translation, and by revisiting households in the evening when the heads of household were more likely to be present (Line 577-586).

9. Data Collection & Quality Control: How was the accuracy of iodine measurement ensured, particularly for the WYD machine analysis.

**Response to the reviewer:** Before quantitative analysis, a measurement quality check was performed in Maweni RRH laboratory to ensure the accuracy and reliability of the instruments (Line 228-230).

10. Data Collection & Quality Control: Can the authors clarify whether the same research assistants collected both qualitative and quantitative data? If so, were they trained to avoid interviewer bias.

**Response to the reviewer:** The qualitative part was conducted by the Principal investigator; the research assistant only helps on Quantitative part (Line 201-204).

11. Data Analysis: What measures were taken to address missing data, if any?

**Response to the reviewer:** Our study had complete data, with no missing values observed during analysis. This was ensured through the thorough training of research assistants and daily supervision by the principal investigator, who carefully reviewed all forms for completeness and accuracy.

12. Data Analysis: Why was a p-value threshold of 0.2 used for bivariable analysis inclusion?

**Response to the reviewer:** A p-value threshold of 0.2 was used for inclusion of variables from the bivariable analysis into the multivariable model to ensure that potentially important variables were not prematurely excluded. This more relaxed threshold compared to the conventional 0.05 allows for the identification of variables that may not show strong individual associations with the outcome but could become significant when adjusted for other variables in the multivariable context.

Using a higher p-value (e.g., 0.2) is a common practice in epidemiological research and model building to avoid missing potential confounders or predictors due to the masking effect of other variables. Stricter thresholds (like 0.05 or 0.1) might lead to the exclusion of variables that contribute meaningfully to the model when assessed jointly.

13. Data Analysis: Were any adjustments made for clustering in the two-stage sampling approach?

**Response to the reviewer:** Yes, the design effect was used to adjust for Clustering in the two-stage sampling (Line 156-157).

14. Ethical Considerations: How was confidentiality maintained during in-depth interviews, considering the sensitivity of some responses

**Response to the reviewer:** All the participants were given a unique ID number, during the interview no name was asked to any participant and all the interviewed participant were followed to their respective farms and asked individually not in groups (Line 307-309).

15. Ethical Considerations: Were participants informed about their right to withdraw from the study at any time without consequences.

**Response to the reviewer:** Yes, this was done and all participants were given informed consent that highlighted the right to withdraw from the study any time without consequences (Line 307-310).

16. Clarity and Rationale: The study highlights a significantly low proportion (9.4%) of households utilizing adequately iodized salt. Could the authors provide more context on national or regional policies regarding salt iodization and enforcement?

**Response to the reviewer**: There is a legal framework under the Tanzania Food, Drugs and Cosmetics Act (CAP 219), specifically the Iodated Salt Regulations, published on 23/04/2010 which governs the production, importation, and distribution of iodated salt in the country. These regulations outline several key requirements, including: restrictions on the importation of iodated salt; standards for edible salt; conditions for packaging and labeling; compliance requirements for packaging, storage, inspection, and analysis of iodated salt by authorized officers; as well as penalties for non-compliance added in manuscript (line 430-436.)

17. The discussion compares findings to various studies in Ethiopia and India. Why were these specific regions chosen for comparison? Are there studies within Tanzania or East Africa that might offer more relevant comparisons?

**Response to the reviewer:** There were no specific region chosen for comparison, Ethiopia and India was chosen because majority of the studies concerning factors associated with utilization of adequately iodized salt was found there as compared to any other regions.

18. Methodology: What was the sampling technique used to select the 435 households? Was it random sampling, stratified sampling, or another method.

**Response to the reviewer:** Systematic sampling technique was used for the selection of 493 households from 4 selected wards (Line 162-167).

19. Methodology: The study mentions binary and multivariable logistic regression analyses. Could the authors clarify if they conducted any model diagnostics (e.g., goodness-of-fit tests) to assess the reliability of their findings.

**Response to the reviewer:** We examined variance for all variables included in the multivariable model to detect multicollinearity.

20. Methodology: The manuscript states that cultural beliefs were associated with salt utilization in the binary regression but were not significant in the multivariable model. Could the authors elaborate on potential confounders that might have led to this change in significance.

**Response to the reviewer:** A possible confounder that may have influenced this change is the availability of iodized salt in the community. Regardless of participants' responses, if the salt commonly available in the area is iodized or not, it could affect the outcome.

21. Findings and Interpretation: The study identifies a strong association between salt storage conditions and iodine retention. Were other household practices, such as cooking methods (e.g., adding salt before or after boiling), considered as potential factors?

**Response to the reviewer:** Thank you for the comment, however cooking practice was not assessed.

22. Findings and Interpretation: The role of knowledge about iodized salt is emphasized in the discussion. Did the study assess the sources of knowledge (e.g., health campaigns, media, school education) to better understand how information dissemination could be improved?

**Response to the reviewer:** Thank you for the comment, we have added informations on knowledge that were not included in this manuscript to enhance our findings (Line 351-358 and table 2).

23. Limitations and Recommendations: Were there any limitations regarding data collection, such as social desirability bias, especially in self-reported knowledge about iodized salt

**Response to the reviewer:** Yes, we encountered three limitations during data collection: unavailability of the head of household at home during the day, language barriers, and social desirability bias (Line 577-586).

24. Limitations and Recommendations: The authors recommend working with local salt producers to ensure a reliable iodized salt supply. Have there been any past interventions in Tanzania to improve iodized salt availability, and how effective were they.

**Response to the reviewer:** Thank you for the comment, Yes, Tanzania has implemented several strategies to improve iodized salt availability (Line 451-458).

**Recommendations for Improvement:**

1. Clarify Study Area Selection: Provide more details on why Kilwa was considered the "worst-performing" district in Lindi beyond citing an unpublished TFNC report. If possible, reference publicly available data.

**Response to the reviewer:** Apart from the unpublished TFNC quarterly report, no other data source was used to justify the selection of Kilwa District. The TFNC report was utilized based on the assumption that it is an official document used by the Tanzania Food and Nutrition Centre for decision-making purposes (Line 142-144).

2. Improve Sampling Justification: Explain why different selection intervals were used for different wards.

**Response to the reviewer:** Different selection intervals were used for different wards to account for variations in population size within each ward. This approach ensured proportional representation across wards, allowing for a more balanced and equitable sample distribution. By adjusting the selection interval based on the total number of households per ward, the sampling process-maintained consistency in coverage while avoiding over- or under-representation of any specific area (Line 167-176).

3. Improve Sampling Justification: Justify the choice of convenience sampling for local production sites and discuss potential limitations.

**Response to the reviewer:** Convenience sampling was employed to select local iodized salt production sites due to its practicality, ease of access, and time efficiency. This method allowed the researchers to gather data from sites that were readily available, its hamlet leader was present in a day of interview and willing to participate. The choice was also influenced by logistical constraints, such as limited funding and time, as well as the absence of a complete list or registry of all production sites in the area. However, this approach has potential limitations. It may introduce selection bias, as the selected sites might not be representative of all salt producers in the region. Consequently, the findings may have limited generalizability, reducing their applicability to the broader population. Despite these limitations, convenience sampling was considered appropriate for the exploratory nature of this study and for generating initial insights into the local production and availability of iodized salt (Line 174-187).

4. Enhance Methodological Transparency: Provide more information on how qualitative data saturation was determined.

**Response to the reviewer:** Qualitative data saturation for the in-depth interviews was determined after conducting nine out of the planned fourteen interviews, no new information was emerging. At this point, participant responses became repetitive and consistent with previously gathered data, indicating that thematic saturation had been reached. Although three additional interviews were conducted to confirm and strengthen the identified information, they did not yield any new information. This confirmed that the data collected after the ninth interview was sufficient to capture the range of perspectives relevant to the study objectives (Line 187-195).

5. Enhance Methodological Transparency: Clarify whether data collectors were blinded to the iodine content of the salt samples to reduce potential bias.

**Response to the reviewer:** The data collectors did not know the iodine content (whether it was high, low, or absent) of the salt samples they were collecting or testing. They were blinded, they treat all samples equally and record exactly what the test shows no assumptions (Line 204-207).

6. Strengthen Data Analysis Explanation: Explain whether and how potential confounders were accounted for in the logistic regression model.

**Response to the reviewer:** A possible confounder that may have influenced this change is the availability of iodized salt in the community. Regardless of participants' responses, if the salt commonly available in the area is iodized or not, it could affect the outcome.

7. Strengthen Data Analysis Explanation: Provide a rationale for why NVivo was chosen for qualitative data analysis over other possible methods.

**Response to the reviewer:** NVivo was chosen because it is user-friendly, there was access to experts who could provide support during the analysis process, and we preferred the use of commercial software. (Line281-283)

8. Address Ethical and Practical Considerations: Explicitly state whether informed consent was obtained from all participants for audio recording.

**Response to the reviewer:** Informed consent was obtained for both quantitative and qualitative data collection (Line 307-310).

9. Address Ethical and Practical Considerations: Discuss how potential risks to participants were minimized, especially for local salt producers.

**Response to the reviewer:** To ensure ethical standards were upheld, several measures were taken to minimize potential risks to participants, particularly local salt producers. Participation was entirely voluntary, and informed consent was obtained from all individuals prior to data collection. Participants were assured that their responses would remain confidential and that no identifying information would be included in any reports or publications. Interviews were conducted in a respectful and non-intrusive manner, ensuring that participants felt comfortable sharing their views. For local salt producers, care was taken to clarify that the study was not regulatory in nature and that the information collected would not be used for enforcement or inspection purposes. This helped reduce any fear of reprisal or negative consequences. Additionally, data collection on local salt producers was done by Principal investigator that had knowledge to uphold ethical principles, including respect, privacy, and cultural sensitivity throughout the study process (Line 313-322).

10. Data Presentation & Clarity: The manuscript refers to Figure 2 but does not describe it in detail. A more explicit explanation of the figure’s content would improve readability.

**Response to the reviewer:** The manuscript has been updated to include a more explicit explanation of the figure's content for better clarity (Line 343-349).

11. Methodology Enhancement: If feasible, provide details on how "good knowledge" about iodized salt was assessed. Was there a standardized questionnaire or knowledge scale used?

**Response to the reviewer:** In this study Knowledge was assessed by calculating the mean score from four sets of questions designed to evaluate participants’ knowledge by assigning 1 point to each correct answer, and 0 to an incorrect answer. First, the total number of correct answers was calculated for each participant. Then, the mean score was obtained by summing the correct answers of all participants and dividing by the total number of participants. Participants who scored greater than or equal to the mean were classified as having good knowledge, while those who scored below the mean were classified as having poor knowledge (Line 256-265).

12. Further Discussion and Implications: Given the cultural preference for non-iodized salt, it would be useful to suggest culturally sensitive education strategies that address local misconceptions.

**Response to the reviewer:** The manuscript has been updated to include a suggestion on implementing culturally sensitive education strategies aimed at addressing local misconceptions about iodized salt (Line558-569).

13. Further Discussion and Implications: The study highlights financial barriers to iodized salt use. Can the authors discuss possible government subsidies or pricing strategies to improve affordability?

**Response to the reviewer:** Yes, the discussion on government strategy to increase affordability has been included in discussion (Line 451-458).

14. Policy and Public Health Recommendations:
a) Advocate for stricter enforcement of salt iodization policies, similar to Nepal's Iodized Salt Act, as a potential regulatory approach for Tanzania
b) Suggest community-level initiatives, such as engagement with village health workers, to educate and promote adequately iodized salt utilization

**Response to the reviewer:** Thank you for the suggestions, the recommendations have been in cooperated in the manuscript (Line 602-605).

**Reviewer No. 3**

**Comments/concerns/suggestions by reviewer 3:**

1. What is the actual message is not clearly identified?

**Response to the reviewer:** We appreciate the Comment. We acknowledge that the main message of the manuscript was not clearly articulated in the previous version. In response, we have revised the introduction, results, and conclusion sections to clearly emphasize the central message of the study.

2. Methods section is very weak

**Response to the reviewer:** We appreciate the Comment: The Methods section was revised to include clearer explanations and newly added subsections for better organization.

3. What is the meaning of WYD machine?

**Response to the reviewer:** The meaning of WYD machine has been included in the manuscript at definition of terms section (line 303).

4. In 201 no line Mean or median could be included

**Response to the reviewer:** Thank you for your observation. We have reviewed the section and agree that including a measure of central tendency would enhance the clarity and interpretation of the data. We have now added the mean and its standard deviation in our manuscript (line 330-331).

5. In table 1, in which basis income level is classified?

**Response to the reviewer:** Thank you for your comment, Income level was classified based on data from the **Household Budget Survey (HBS)** conducted by the National Bureau of Statistics (NBS) to assess income levels and poverty. This survey collects detailed information on household income and expenditure, which helps in understanding the distribution of income across different regions and demographics (Line 265-269)

6. In 209 line, in laboratory survey, it was found only 91 sample was collected for idodine analysis. Why it is 435? Please justify.

**Response to the reviewer:** In line 209, it should read 41 samples instead of 91 samples. Our study had a binary outcome: utilization of adequately iodized salt versus utilization of inadequately iodized salt. Initially, all salt samples were qualitatively tested for iodine using an iodine rapid testing kit. A total of 89 samples changed to blue or purple upon exposure to the test solution and were thus considered iodized. The remaining 394 samples did not show a color change and were categorized as non-iodized; these were not sent for further laboratory analysis.The 89 iodized samples were subjected to quantitative analysis using the WYD machine. Of these, only 41 samples had an iodine content of ≥15 ppm and were classified as adequately iodized. The 394 non-iodized samples and the 48 iodized samples with iodine content <15 ppm were all categorized as inadequately iodized.

7. In Table 2, In excel sheet data entries 477, but the sample size is 435. what is the reason

**Response to the reviewer:** We appreciate the reviewer’s observation and the opportunity to clarify. During the data cleaning process, we identified that some participants were mistakenly collected from wards that were not part of our designated study area. As a result, 42 entries were excluded from the final analysis to ensure consistency with our study design and inclusion criteria.We acknowledge that the Excel sheet initially shared was not the final cleaned version, and we apologize for this oversight. The updated and cleaned dataset reflecting the correct sample size of 435 has now been submitted

8. In Table 2, What were their results on Income source, Income level, Salt preference.

**Response to the reviewer:** Thank you for your comment. In Table 2, we presented only the variables that showed a statistically significant association with the outcome in the multivariable logistic regression analysis. Income source, income level, and salt preference were included in the initial analysis but did not demonstrate a significant association with the utilization of adequately iodized salt. As a result, they were excluded from the final table to maintain clarity and focus on the most relevant findings.

9. In Table 2, how can you explore the cultural beliefs?

**Response to the reviewer:** Thank you for your comment. Cultural beliefs and how to explore them have been added to our manuscript (lines 249–251).

10. In Table 2, all this variable should describe in data collection instrument development section. In which basis the categorize the knowledge?

**Response to the reviewer:** Thanks for your observation, description of variables and how knowledge was categorized was added in the manuscript line 259-269.

11. In 411 line, re-write with focus on study finding. Recommendation is not match with study finding.

**Response to the reviewer:** Thank you for your suggestion. The recommendations have been revised to align with the study findings.
