# Supplementary material for: Utilization of adequately iodized salt and its associated factors in Tanzania rural areas: a case of Kilwa district, Lindi region, 2023
Source: PLoS One. 2025 Nov 24;20(11):e0337337. doi: 10.1371/journal.pone.0337337 (PMC12643270; doi:10.1371/journal.pone.0337337)
Supplement: Attachment — Submitted filename: Response to editors comments.docx [file pone.0337337.s007.docx]

**Dear Editor,**

We express our gratitude to the editor for their valuable comments, which have significantly improved our manuscript titled **"** **Utilization of adequately iodized salt and its associated factors in Tanzania rural areas: a case of Kilwa district, Lindi region, 2023".** We have carefully revised our manuscript addressing all comments and suggestions provided by the editor. We believe that we have effectively addressed the raised concerns and that, after incorporating the changes, our manuscript is now appropriate for publication.

Kind regards,

**Dr. George Mrema**

**Comments/concerns/suggestions by Editor:**

1. In the introduction, a number of places need to improvement. Firstly, the objective must be clearly stated as the very last sentence " To supplement existing literature, therefore, this current work assessed household utilization of adequately iodized salt and its associated factors in Kilwa district, which is among Tanzania rural areas, and this was performed by way of cross-sectional design across local salt producers" (Please insert this sentence in current line 86).

**Response to the editor:** We appreciate the editor's comments. The suggested sentence has been incorporated into the manuscript at line 132.

2. Now, let's look at the overall introduction. It is not yet complete. Merge line 48-63 as one paragraph. Also, merge lines 77-86 as one paragraph. This will give 4 paragraphs in total. Before talking about USI in Tanzania, please, create a new paragraph to talk about salt production process in Tanzania. Tell us the steps of salt production, tell us the number of salt makers in Tanzania, tell us how their packaging looks like, tell us how many people generally work in a typical local salt producer, tell us their pattern of salt product distribution, tell us about affordability of salt (sources of this information could be newspapers, government bulletins, personal communication), I expect this will likely be one strong paragraph, at the maximum two strong paragraphs.

**Response to the editor:** Thank you so much for the comment. All suggested revisions have been incorporated into the manuscript, specifically in lines 48–100.

3. Go to the methods, change the title of "2. Study methodology" (not "Materials and methods"). Now, begin this section by creating a new subsection called "Schematic overview of study program", which would comprise 5 sentences, and supported compulsorily by a flow diagram that shows the major methodology steps that built the entire study. Sentence one must introduce the flow diagram, sentence two briefly outlines the major stages, sentence 3 links the flow diagram with the objective of this work, sentence 4 tells us why this approach is unique, sentence 5 tells us how these steps were validated.

After this section called "Schematic overview of study program", the next section must be "Questionnaire development process". Tell us, how did you develop the questionnaire (the research instrument)? how did you use specialist experience/expertise and knowledge together with synthesised relevant literature to come up with the questionnaire items? How did you develop the questionnaire contents, and did you do content validation? After the validation process, who subsequently approved the questionnaire? Is it the association body for the local salt producers (just curious)? Was there any prerequisite conditions set out for questionnaire participation? Tell us about the arrangement of the actual questionnaire, how many pages, how many items in total, and the major sections. (Make sure to include aspects of this in the flow diagram, and mention it tinly in the " "Schematic overview of study program" section). How did you differentiate between Semi Structured and pre-tested questionnaires? what was the main purpose to do so?

**Response to the editor:** We thank the editor for pointing out this. All suggested comments have been addressed and incorporated into the manuscript, specifically in lines 137–170.

4. Please amend "Study design, area and population under the study" to "Study design, area and population". The rest of the 2. Study methodology" is ok.

**Response to the editor:** We appreciate the editor's suggestion. The manuscript has been revised accordingly to address the comment, specifically on line 171.

5. Results and discussion are excellent. Please, in the discussion, make best effort to capture all the tables mentioned in the results. Use "(Refer to Table ?) in all the places where data is each table is discussed

**Response to the editor:** We appreciate the editor’s suggestions. Accordingly, we have referred to the relevant table numbers in the discussion section (Lines 456–601) to enhance clarity and improve linkage for the readers

6. Before conclusion, I believe that there were limitations that you encountered, please create a new section, "Challenges and limitations of current study", make sure to brainstorm on limitations you encountered in developing the questionnaire, developing the entire methods, assembling the data, analyzing the data, discussing the findings, logistical challenges, administrative challenges, etc. Maximum, two paragraphs. Authors apply your kind discretion to develop this section.

**Response to the editor:** We thank the editor for pointing out this. Changes have been made to incorporate these revisions (lines 602–628).

7. In the conclusion, provide clear direction for future study. Also, what is the take home lesson for policy makers and other stakeholders.

**Response to the editor:** We appreciate editor’s suggestions. Conclusion section was revised to provide clear direction and lesson for police makers (Line 660-674).
