# Supplementary material for: Utilization of adequately iodized salt and its associated factors in Tanzania rural areas: a case of Kilwa district, Lindi region, 2023
Source: PLoS One. 2025 Nov 24;20(11):e0337337. doi: 10.1371/journal.pone.0337337 (PMC12643270; doi:10.1371/journal.pone.0337337)
Supplement: Attachment — Submitted filename: Response_to_reviewers_comments_auresp_3.docx [file pone.0337337.s008.docx]

**Dear Editor,**

We express our gratitude to the editor and reviewers for their valuable comments, which have significantly enhanced our manuscript titled **"** **Utilization of adequately iodized salt and its associated factors in Tanzania rural areas: a case of Kilwa district, Lindi region, 2023".** We have considered all the comments and integrated their suggestions into the revised version of the manuscript. We believe that we have effectively addressed the raised concerns and that, after incorporating the changes, our manuscript is now appropriate for publication.

Kind regards,

**Dr. George Mrema**

**Reviewer No. 3**

**Comments/concerns/suggestions by reviewer 3:**

1. This is quite a good research report. Although the study was conducted on a small scale, it is meaningful and necessary for many regions in African countries where people’s awareness of the importance of iodized salt in food remains low. The study also collected a relatively large sample size, sufficient for analysis and assessment of iodized salt utilization in the research area. The information presented in the supplementary materials is also quite interesting and diverse. The methodology and data analysis methods applied are appropriate.
   However, the research results have not been evaluated and discussed from multiple perspectives, which shows that the study has not fully exploited the data collected. In particular, the information collected from 4 wards (namely: Kiranjeranje, Mandawa, Miteja, and Tingi), further divided into 12 villages (namely: Kiranjeranje, Kiswele, Mbwemkuru, Mtandi, Mandawa, Mitumba, Hotel tatu, Kiwawa, Sinza, Matandu, Kibaoni, Njia nne, and Mtandango), has not been analyzed in detail at the smaller administrative unit level.

**Response to the reviewer:** We appreciate the reviewer's comments. We have analyzed our data in detail at the smaller administrative unit level refer to table 1, Fig 3 and 4 (Line 341-345 & 360-367)

.

2. The study results are presented only in Figure 1, which is too simple and sketchy. The paper should include more results in the form of charts to demonstrate thorough data processing, especially results related to educational level, gender, age, and iodized salt usage

**Response to the reviewer:** Thank you very much for your valuable comments and suggestions. In response, we have enhanced the presentation of our results to ensure greater clarity and visual appeal. In addition to the information presented in Table 1, which details demographic characteristics such as educational level, gender, and age, we have now included four additional charts/figures that illustrate the adequate utilization of iodized salt across key demographic variables. These visual representations complement the tabulated data and provide a clearer overview of the distribution and patterns observed in the study area (Line 360-376, Figure 3, 4, 5 and 6).

3. I have some concerns regarding the proportion of survey respondents. The age group 26–35 accounted for a very large number of respondents (252), while other age groups were underrepresented. In particular, the group under 25 years old was very limited (only 16 respondents under 18 and 28 respondents aged 18–25). This group is crucial for nutritional development and represents the future of the country, so such a small number of responses may lead to results that are not truly representative.

**Response to the reviewer:** We thank the reviewer for pointing out this. The study included heads of households who were present at their homes during data collection. The age groups highlighted reflect the participants accessible and available during our survey.

4. Could you clarify whether, among the survey participants, there were any individuals suffering from iodine deficiency-related diseases?

**Response to the reviewer:** We sincerely thank the reviewer for this important question. The present study focused primarily on assessing the utilization of adequately iodized salt and identifying factors influencing its use at the household level. Information on iodine deficiency-related diseases among participants was not collected, as this was beyond the study’s scope.

5. I believe that education is also an important aspect, which was mentioned at the beginning of the paper but not discussed in the conclusion. If possible, a separate section on policy recommendations for the government to improve iodized salt utilization should be developed, which would make the paper much more practically valuable

**Response to the reviewer:** Thank you for your valuable suggestion. We have incorporated additional policy recommendations to assist the government in addressing this issue. Additionally, an education recommendation has been included (Line 628-638).
