# Supplementary material for: Utilization of adequately iodized salt and its associated factors in Tanzania rural areas: a case of Kilwa district, Lindi region, 2023
Source: PLoS One. 2025 Nov 24;20(11):e0337337. doi: 10.1371/journal.pone.0337337 (PMC12643270; doi:10.1371/journal.pone.0337337)
Supplement: Attachment — Submitted filename: PLOS ONE REVIEWER COMMENTS.docx [file pone.0337337.s003.docx]

This manuscript reports on the "Utilization of adequately iodized salt and its associated factors in Tanzania rural areas: a case of Kilwa district, Lindi region, 2023." While some findings align with public health data, significant revisions are necessary.

**Comments and recommendations for the author:**

**Abstract:**

(a) Your abstract should adhere to the journal's format, presented as a single continuous paragraph while incorporating all necessary components

**Introduction:**

(a) Please ensure consistency in the WHO iodine intake recommendation unit—whether it is in micrograms or grams. Kindly review and revise the reference statement accordingly in lines 51-54 and throughout the manuscript.

**Study Design & Justification:**

(a) Why was a cross-sectional design chosen for this study, and how does it adequately address the study objectives
(b) Were any potential biases considered in selecting the Kilwa district as the study area

**Sampling Methodology:**

(a) How was the sample size of 493 households determined? Were design effects or response rates considered
(b) Can the authors provide more justification for the choice of systematic sampling and how it ensures representativeness
(c) What steps were taken to minimize selection bias, particularly for the qualitative component

**Data Collection & Quality Control:**

(a) Were there any challenges encountered during data collection, and how were they mitigated
(b) How was the accuracy of iodine measurement ensured, particularly for the WYD machine analysis
(c) Can the authors clarify whether the same research assistants collected both qualitative and quantitative data? If so, were they trained to avoid interviewer bias

**Data Analysis:**

(a) What measures were taken to address missing data, if any
(b) Why was a p-value threshold of 0.2 used for bivariable analysis inclusion
(c) Were any adjustments made for clustering in the two-stage sampling approach

**Ethical Considerations:**

(a) How was confidentiality maintained during in-depth interviews, considering the sensitivity of some responses
(b) Were participants informed about their right to withdraw from the study at any time without consequences

**Clarity and Rationale**

(a) The study highlights a significantly low proportion (9.4%) of households utilizing adequately iodized salt. Could the authors provide more context on national or regional policies regarding salt iodization and enforcement?
(b) The discussion compares findings to various studies in Ethiopia and India. Why were these specific regions chosen for comparison? Are there studies within Tanzania or East Africa that might offer more relevant comparisons

**Methodology**

(a) What was the sampling technique used to select the 435 households? Was it random sampling, stratified sampling, or another method
(b) The study mentions binary and multivariable logistic regression analyses. Could the authors clarify if they conducted any model diagnostics (e.g., goodness-of-fit tests) to assess the reliability of their findings
(c) The manuscript states that cultural beliefs were associated with salt utilization in the binary regression but were not significant in the multivariable model. Could the authors elaborate on potential confounders that might have led to this change in significance

**Findings and Interpretation**

(a) The study identifies a strong association between salt storage conditions and iodine retention. Were other household practices, such as cooking methods (e.g., adding salt before or after boiling), considered as potential factors
(b) The role of knowledge about iodized salt is emphasized in the discussion. Did the study assess the sources of knowledge (e.g., health campaigns, media, school education) to better understand how information dissemination could be improved

**Limitations and Recommendations**

(a) Were there any limitations regarding data collection, such as social desirability bias, especially in self-reported knowledge about iodized salt
(b) The authors recommend working with local salt producers to ensure a reliable iodized salt supply. Have there been any past interventions in Tanzania to improve iodized salt availability, and how effective were they

**Recommendations for Improvement:**

1. **Clarify Study Area Selection:**

- Provide more details on why Kilwa was considered the "worst-performing" district in Lindi beyond citing an unpublished TFNC report. If possible, reference publicly available data.

2. **Improve Sampling Justification:**

- Explain why different selection intervals were used for different wards.
- Justify the choice of convenience sampling for local production sites and discuss potential limitations.

3. **Enhance Methodological Transparency:**

- Provide more information on how qualitative data saturation was determined.
- Clarify whether data collectors were blinded to the iodine content of the salt samples to reduce potential bias.

4. **Strengthen Data Analysis Explanation:**

- Explain whether and how potential confounders were accounted for in the logistic regression model.
- Provide a rationale for why NVivo was chosen for qualitative data analysis over other possible methods.

5. **Address Ethical and Practical Considerations:**

- Explicitly state whether informed consent was obtained from all participants for audio recording.
- Discuss how potential risks to participants were minimized, especially for local salt producers.

6. **Data Presentation & Clarity**

- The manuscript refers to Figure 2 but does not describe it in detail. A more explicit explanation of the figure’s content would improve readability.

7. **Methodology Enhancement**

- If feasible, provide details on how "good knowledge" about iodized salt was assessed. Was there a standardized questionnaire or knowledge scale used?

8. **Further Discussion and Implications**

- Given the cultural preference for non-iodized salt, it would be useful to suggest culturally sensitive education strategies that address local misconceptions.
- The study highlights financial barriers to iodized salt use. Can the authors discuss possible government subsidies or pricing strategies to improve affordability?

9. **Policy and Public Health Recommendations**

- Advocate for stricter enforcement of salt iodization policies, similar to Nepal's Iodized Salt Act, as a potential regulatory approach for Tanzania.
- Suggest community-level initiatives, such as engagement with village health workers, to educate and promote adequately iodized salt utilization.
